# Resistance to novel antimicrobial agents among NDM-and OXA-48-producing Carbapenem-resistant Enterobacterales in Lebanon

**DOI:** 10.3389/fcimb.2026.1938955

**Published:** 2026-09-24

**Authors:** George F. Araj, Joseph M. Matar, Chaza K. Al Ghor, Ghena M. Sobh, Clara R. Dagher

**Affiliations:** 1 Clinical Microbiology Laboratory, Department of Pathology and Laboratory Medicine, American University of Beirut Medical Center, Beirut, Lebanon; 2 Center for Infectious Diseases Research, American University of Beirut Medical Center, Beirut, Lebanon; 3 Department of Internal Medicine, American University of Beirut Medical Center, Beirut, Lebanon

**Keywords:** antimicrobial resistance, carbapenem- resistant Enterobacterales, carbapenemases, NG-Test CARBA-5, Novel antibiotics

## Abstract

**Background:**

Rapid detection of carbapenemases and antimicrobial susceptibility testing (AMST) against carbapenem-resistant (CR) Enterobacterales (CRE) are key elements in controlling their nosocomial spread and useful to guide treatment of patients infected with such morbid pathogens. The absence of comprehensive data in Lebanon prompted this study.

**Objective:**

This study aimed at revealing the carbapenemase types among CR isolates recovered from patients representing 100 *Escherichia coli* (CREc) and 40 *Klebsiella pneumoniae* (CRKp). Their antimicrobial resistance (AMR) profiles against routine and novel antimicrobial agents were also determined.

**Methods:**

Identification of isolates was done by MALDI-TOF, and the types of carbapenemases were determined by the rapid NG-Test CARBA. Antimicrobial resistance (AMR) profiles were revealed against the routine antibiotics, including ceftazidime-avibactam, and novel antimicrobial agents that still have not been introduced in this country, namely, imipenem-relebactam, meropenem-vaborbactam, and cefiderocol using disk diffusion and standard Etest methods.

**Results:**

Among both CREc and CRKp, the detected carbapenemases were only NDM and OXA-48, whereby the majority were NDM. The activity of novel antimicrobial agents was higher against CREc than CRKp isolates, and against OXA-48- producing strains than NDM-producing strains in both species.

**Conclusion:**

This first comprehensive *in vitro* study from Lebanon revealed high resistance (28%-100%, and 0%-100% among NDM and OXA-48 producers, respectively) of yet-to-be- introduced novel antimicrobial agents in this country. This highlights the necessity of scrutinizing the proper use of antimicrobial agents, stewardship programs, and infection control aspects to curb the morbid impact of these pathogens.

## 1 Introduction

Carbapenem-resistant (CR) pathogens continue to pose serious and growing challenges at multiple levels, affecting patients, healthcare systems, and public health worldwide. CR Enterobacterales (CRE), also known as carbapenemase-producing Enterobacterales (CPE), are the most frequently encountered among these critical multidrug-resistant (MDR), extensively drug-resistant (XDR), or even pan drug-resistant (PDR) pathogens (Cai et al., 2022; Caliskan-Aydogan and Alocilja, 2023; Daaboul et al., 2023).

Globally, including Lebanon, the prevalence of the CRE pathogens has seen a dramatic rise over the past few years (Araj et al., 2018; Rima et al., 2022; Macesic et al., 2025).

The threat and challenge posed by these pathogens are based on their acquirement of several resistance mechanisms. These include the loss of certain outer membrane porins; the activation of efflux pumps, preventing carbapenems from reaching their site of action; and the acquisition of enzymes like carbapenemases to degrade or hydrolyze carbapenems and other β-lactam antibiotics (Caliskan-Aydogan and Alocilja, 2023).

Carbapenemase production ranks the most prevalently encountered resistant mechanism. Based on their molecular and biochemical composition, they have been classified by Ambler into the following classes: Class A [e.g., *K*. *pneumoniae* carbapenemases (KPC)], Class B [encodes metallo-β-lactamase enzymes (MBL), including New Delhi metallo-β-lactamase (NDM), Verona integron-encoded metallo-β-lactamase (VIM), and imipenemases encoding metallo-β-lactamase (IMP)], and Class D [e.g., oxacillinases carbapenemases (mainly OXA-48), which are serine-β-lactamases]. Among these, five carbapenemase types top the list: NDM, OXA-48, VIM, IMP, and KPC (Papp-Wallace et al., 2011). In this study, all OXA subtypes will be thereafter cited as OXA.

The rapid detection of these carbapenemase types would be a key element in controlling nosocomial spread as well as useful to tailor and guide physicians towards proper treatment of patients infected with such pathogens (Zhang et al., 2022). Such treatments include novel antimicrobial agents such as ceftazidime-avibactam (CZA), imipenem-relebactam (Imp-R), meropenem-vaborbactam (Mer-V), and cefiderocol (CFD). To achieve these endeavors, quite a few rapid phenotypic and molecular methods are available (O'Donnell and Lodise, 2022). As such, several studies around the world have reported on the prevalence of carbapenemase types involved in CREs in their countries. In Lebanon, despite the growing burden of CRE the local evidence on the involved carbapenemase types remain scarce, and no comprehensive susceptibility studies have been reported. This major gap limits the ability to guide antimicrobial stewardship, prompting this study to investigate the prevalence of carbapenemase types among CR *Escherichia coli* (CREc) and *Klebsiella pneumoniae* (CRKp) isolates, as well as to characterize their antimicrobial resistance profiles against conventional and novel antimicrobial agents that have not yet been introduced in the country.

## 2 Materials and methods

### 2.1 Inclusion criteria

This study included confirmed CRE isolates of *E*. *coli* and *K*. *pneumoniae* producing NDM and OXA-48 carbapenemases.

### 2.2 Exclusion criteria

Isolates other than *E*. *coli* and *K*. *pneumoniae*, isolates producing carbapenemases other than NDM or OXA-48, and duplicate isolates recovered from the same patient were excluded.

### 2.3 Bacterial isolates

Non-duplicate CR *E*. *coli* (n=100) and CR *K*. *pneumoniae* (n= 40) isolates were collected from different clinical samples submitted for culture at the Clinical Microbiology Laboratory, Department of Pathology and Laboratory Medicine, American University of Beirut Medical Center, from January 2024 to February 2025.

### 2.4 Identification

Identification of the isolates was done by using the matrix-assisted laser desorption ionization-time of flight mass spectrometry (MALDI-TOF) with its MALDI-biotyper software (Bruker Daltonics, Bremen, Germany).

### 2.5 Screening and confirmation of carbapenem-resistant isolates

Screening of isolates for carbapenem resistance was done using the disk diffusion (DD) method, applying the ertapenem disk (10 µg). To confirm the suspected resistant findings, the minimal inhibitory concentrations (MICs) of ertapenem, imipenem, and meropenem were determined using the E-test methodology (AB BIODISK, Solna, Sweden) as was reported from our lab earlier (Araj et al., 2018). Interpretation of the susceptibility category was done following the CLSI guidelines (Clinical and Laboratory Standards Institute, 2023).

The DD method was also used for routinely tested antimicrobial agents against carbapenem-resistant isolates. Commercial disks and their concentration (BBL, Becton Dickinson, USA) were used, as follows: amikacin (30 µg), gentamicin (10 µg), ciprofloxacin (5 µg), piperacillin/tazobactam (100/10 µg), trimethoprim/sulfamethoxazole (1.25/23.75 µg), tigecycline (15 µg), and fosfomycin/trometamol (200 μg).

### 2.6 Detection of carbapenemases

Carbapenem-resistant isolates were tested for detecting the type of carbapenamases in each of the isolates using the NG-Test CARBA-5 (Biotech SAS, Guipry, France). It is a visual multiplex rapid immunochromatographic qualitative assay for the detection and differentiation of the 5 most common carbapenemase families: KPC, OXA-48-like, VIM, IMP, and NDM. The test was performed and interpreted according to the manufacturer’s instructions.

### 2.7 Susceptibility testing of novel antimicrobial agents

The testing and interpretation of the susceptibility category of the novel antimicrobial agents against the isolates were done following the CLSI guidelines (Clinical and Laboratory Standards Institute, 2023). The E-test methodology was used to determine the MIC values of CZA, while the disk diffusion methodology was used to determine the inhibition zones of Imp-R, Mer-V, and CFD against the tested isolates.

### 2.8 Statistical analysis

Categorical variables were summarized as frequencies and percentages [n (%)]. Statistical analyses were performed using GraphPad Prism version 8.4 for Windows (GraphPad Software, La Jolla, CA, USA). Differences in antimicrobial susceptibility between NDM- and OXA-producing isolates were assessed separately for *Escherichia coli* and *Klebsiella pneumoniae*. Categorical variables were compared using the chi-square (χ^2^) test when all expected cell counts were ≥5; otherwise, Fisher’s exact test was applied. All tests were two-sided, and a p-value <0.05 was considered statistically significant.

### 2.9 Quality control

Quality control of disk diffusion and MIC methods was ensured using the American Type Culture Collection (ATCC) quality control strains of *E*. *coli* (ATCC 25922) and *Pseudomonas aeruginosa* (ATCC 27853).

## 3 Results

### 3.1 Prevalence of carbapenemases among Enterobacterales

The total number of requested testings for detection and identification of carbapenemases among carbapenem-resistant bacteria was 166, representing eight species, and distributed as presented in **Table 1**. The predominance of NDM carbapenemase was noted among all these species except *Pseudomonas aeruginosa*, where VIM carbapenemases were higher. The latter carbapenemase was also detected in *Enterobacter hormaechei* and *Citrobacter freundii.*

**Table 1:** Distribution of carbapenemases detected among Carbapenem Resistant bacteria requested for CARBA-5 testing.

Bact species	Number (%) of isolates revealing carbapenemases					
	Number tested	OXA-48	NDM	Vim	OXA + NDM	NEG
*Escherichia coli*	100	29 (29)	64 (64)		1 (1)	6 (6)
*Klebsiella pneumoniae*	40	9 (22.5)	23 (57.5)		5 (12.5)	3 (7.5)
Enterobacter hormaechei*	13	2	10	1		
Citrobacter freundii*	3		2	1		
Morganella morganii*	1		1			
Providencia rettgeri*	2		2			
Serratia marcescens*	2		1			1
Pseudomonas aeruginosa*	5		2	3		

NDM, New Delhi metallo-β-lactamase; OXA-48, Oxacillinase-48; Vim, Verona integron-encoded metallo-β-lactamase.

*The absence of citing % to these bacteria is due to their low numbers.

As shown in **Table 1**, CREc (n=100) and CRKp (n=40) were the most commonly encountered isolates, thus warranting analysis in this study. The prevalence of carbapenemase types among these two species, respectively, was NDM 64% and 57.5%, OXA 29% and 22.5%, dual for both NDM plus OXA 1% and 12.5%, and the absence of carbapenemases was 6% and 2.5%.

### 3.2 Demographics

#### 3.2.1 The source of specimen

The specimen sources for these recovered CREc and CRKp isolates were from different sites and recovered in variable frequencies, respectively, the highest being from urine (27% and 35%), skin screening (33% and 30%), DTA (4% and 17.5%), and blood (21% and 10%). The recovery of these species from other specimens such as tissue, wound, fluid, catheter, and bone ranged between 0% and 5%.

The hospital ward/unit distribution of these CRKp and CREc isolates, respectively, was: critical care units (53% and 35%), medical (16% and 27%), oncology (16% and 20%), emergency (9% and 8%), surgical (6% and 10%),

#### 3.2.2 Age

Regarding age, the isolates were arranged in 5-year age ranges starting from newborn to age ≥ 66 years. In those patients with ages ≤ 50, the number of isolates was between 0 and 4 isolates for each age range. The highest prevalence of CREc and CRKp was noted among patients aged ≥ 51 years. The majority (76%) being detected among the age group of ≥ 66 years. NDM carbapenemases predominated over OXA at all ages.

#### 3.2.3 Gender

Taking gender into consideration, the presence of carbapenemase types among males and females are similar for NDM, and with some fluctuation in OXA among both species.

### 3.3 Effect of routinely tested antimicrobial agents

**Table 2** shows the activity of 7 routinely tested antimicrobial agents (amikacin, gentamicin, ciprofloxacin, piperacillin-tazobactam, trimethoprim-sulfamethoxazole, tigecycline, and fosfomycin) against the carbapenemases in both bacterial species. Poor activity (0%-48%) of these drugs was shown against the NDM-producing CRKp. However, tigecycline, Fosfomycin, and amikacin retained good activity (79%-100%) against NDM- and OXA-producing CREc.

**Table 2:** Susceptibility results among routinely tested antimicrobial agents as related to CARBA-5 genes in CRE *E. coli* and *K. pneumoniae.*

Antimicrobial Agent	Number (%) susceptible to routinely tested regarding type of carbapenemases among bacteria					
	*E. coli* (n=100)			*K. pneumoniae* (n=40)		
	NDM (n=64)*	OXA (n=29)*	p-value	NDM (n=23)*	OXA (n=9)*	p-value
Amikacin	57 (89)	29 (100)	0.0942	5 (22)	7 (78)	**0.0057**
Gentamicin	43 (67)	16 (55)	0.2651	7 (30)	8 (89)	**0.0049**
Ciprofloxacin	1 (2)	4 (14)	**0.0315**	0 (0)	1 (11)	0.2912
Piperacillin-tazobactam	0 (0)	0 (0)	>0.9999	0 (0)	0 (0)	>0.9999
Trimethoprim-sulfamethoxazole	1 (2)	9 (31)	**<0.0001**	4 (17)	4 (44)	0.176
No. Susceptible / No. Tested (%)						
Tigecycline^+^	31/32 (97)	12/12 (100)	>0.9999	6/13 (46)	6/6 (100)	**0.0436**
Fosfomycin^+^	49/58 (84)	19/24 (79)	0.5605	10/21 (48)	3/8 (38)	0.6243

*These numbers exclude the isolates of dual NDM+OXA carbapenemases and the Negative isolates.

+ Not all isolates were tested against these antibiotics due to clinical significance based on sites of infection.

P-value ≤0.05 is statistically significant and noted in bold.

### 3.4 Effect of novel tested antimicrobial agents

Regarding novel antimicrobial agents, **Table 3** shows the activity of the routinely tested carbapenems [ertapenem (Ert), imipenem (Imp), and meropenem (Mer)], the novel antimicrobials Imp-R, Mer-V and CFD, as well as that of CZA against CREc and CRKp isolates producing NDM and OXA carbapenemases.

**Table 3:** Susceptibility of carbapenems and novel carbapenems against carbapenem resistant *E*. *coli* and *K*. *pneumoniae* harboring NDM and OXA-48 carbapenemases.

Antimicrobials/susceptibility category		Number (%) findings among isolates with carbapenemases					
		CREc (n=100)			CRKp (n=40)		
		NDM (n=64)*	OXA-48 (n=29)*	P-value	NDM (n=23)*	OXA-48 (n=9)*	P-value
Susceptible	Ertapenem	1 (2)	0 (0)	>0.9999	0 (0)	0 (0)	N/A
	Imipenem	0 (0)	3 (10)	**0.0282**	0 (0)	0 (0)	N/A
	Imipenem-Relebactam	0 (0)	12 (41)	**<0.0001**	0 (0)	1 (11)	0.2812
	Meropenem	0 (0)	7 (24)	**<0.0001**	0 (0)	1 (11)	0.2812
	Meropenem-Vaborbactam	6 (10)	28 (96)	**<0.0002**	1 (6)	5 (55)	**0.0018**
	Cefiderocol	46 (72)	27 (93)	**0.0279**	14 (60)	9 (100)	**0.0348**
	Ceftazidime-Avibactam	0 (0)	25 (86)	**<0.0001**	0 (0)	7 (78)	**<0.0001**
Intermediate Susceptible	Ertapenem	0 (0)	1 (3)	0.3118	0 (0)	0 (0)	N/A
	Imipenem	2 (3)	9 (31)	**0.0004**	0 (0)	1 (11)	0.2812
	Imipenem-Relebactam	3 (5)	14 (49)	**<0.0001**	0 (0)	3 (34)	**0.0125**
	Meropenem	1 (2)	9 (31)	**<0.0001**	0 (0)	0 (0)	N/A
	Meropenem-Vaborbactam	5 (8)	1 (4)	0.6616	1 (5)	0 (0)	>0.9999
	Cefiderocol	13 (21)	2 (7)	0.1341	4 (18)	0 (0)	0.3035
	Ceftazidime-Avibactam	0 (0)	0 (0)	N/A	0 (0)	0 (0)	N/A

S, Susceptible; I, Intermediate Susceptible; IMP-R, Imipenem-relebactam; Mer-V, Meropenem-vaborbactam; CFD, Cefiderocol; CREc, carbapenem resistant Escherichia coli; CRKp, carbapenem resistant Klebsiella pneumoniae; NDM, New Delhi metallo-β-lactamase; OXA-48, Oxacillin carbapenemases; 0, Zero value.

*These numbers exclude the NDM+OXA and the Negative isolates.

Decimals were rounded.

P-value ≤0.05 is statistically significant and noted in bold.

Ert showed nil activity against carbapenemases from all isolates. Imp and the novel Imp-R activities were nil against NDM producing isolates of both species. Their activities against OXA producers in both species were poor (0%-43%). However, they showed intermediate susceptible activity of 31%-49% and 11%-34% against OXA producers in CREc and CR Kp, respectively. Regarding the activity of Mer, it was poor (≤ 24%) against CREc- and CRKp- producing OXA, and almost nil against NDM producers in both species. The activity of Mer-V against OXA producers was excellent (96%) against CREc and low (50%) against CRKp isolates. However, this novel drug activity was almost nil (6%-10%) against NDM producers in both species. CZA activity was reasonably acceptable against OXA producers of both CREc (86%) and CRKp (78%). However, its activity against NDM producers was nil among both species. The novel Cefiderocol showed excellent activity against OXA producers of both CREc (93%) and CRKp (100%). However, its activity against NDM producers in both species was humble: 72% for CREc and 60% for CRKp.

CREc isolates were less resistant than CRKp against CZA and all the three novel antimicrobial agents.

**Figure 1** provides a visual illustration of the novel antimicrobial agents activities against the carbapenemases in both species.

**Figure 1:**
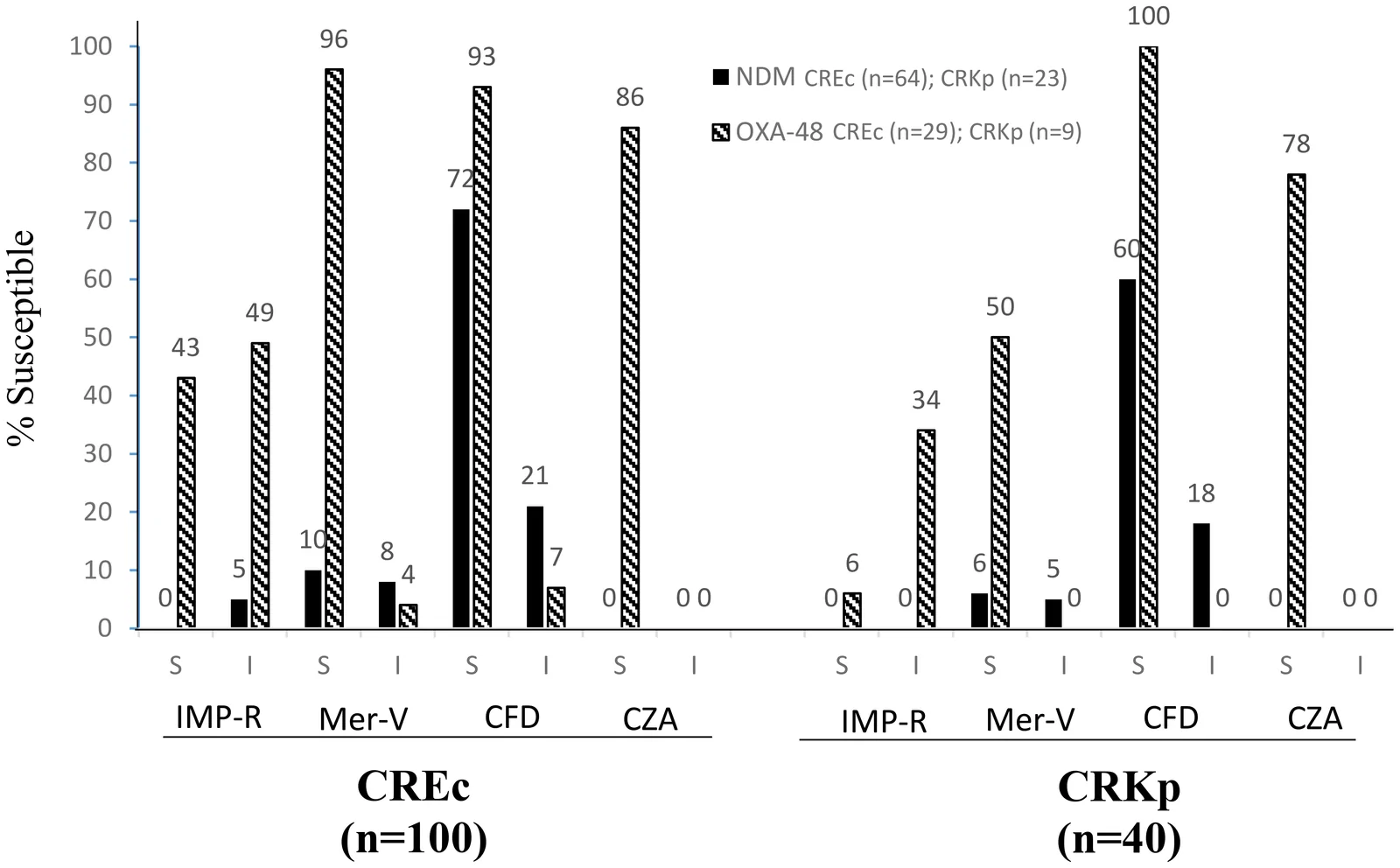
Activity of the novel antibiotics against the detected carbapenemases among CREc and CRKp. S, Susceptible; I, Intermediate Susceptible; IMP-R, Imipenem-relebactam; Mer-V, Meropenem-vaborbactam; CFD, Cefiderocol; CREc, carbapenem resistant *Escherichia coli*; CRKp, carbapenem resistant *Klebsiella pneumoniae*; NDM, New Delhi metallo-β-lactamase; OXA-48, Oxacillin carbapenemases; 0, Zero value.

## 4 Discussion

In Lebanon and, for example, at our medical center, the rates of CREc and CRKp have risen from 0.9% and 4% in 2013 to 5% and 13% in 2022, as reported in the circulated annual “Antimicrobial Resistance of Pathogens” brochure showing susceptibility profiles (https://www.aub.edu.lb/fm/PLM/Pages/ClinicalMicrobiology.aspx). The increase in CR enterobacterales pathogens’ prevalence necessitates their characterization to help guide definite antimicrobial selection for treatment decisions (Xu et al., 2017).

In this context, this current study from Lebanon for the first time provided details on the phenotypic types of carbapenemase produced by both CREc and CRKp clinical isolates. It also reports on the antimicrobial susceptibility profiles of these species against CZA in addition to the novel antimicrobial agents: Imp-R, Mer-V, and CFD. For the clarity of discussion and comparison with other studies published from our region and other countries, the findings will be presented separately regarding each species. In order to make the relevance of findings in our study with those previously reported regionally and from other countries, the prevalence of individual types as well as the combination of more than one type of carbapenemases harbored by CRKp and CREc will be discussed as shown below.

Historically, the prevalence of carbapenemases had a typical geographic distribution: KPC in the USA, Europe, and China; VIM in Greece; OXA in Turkey; and NDM in the Indian subcontinent. Subsequently, strains of carbapenem-resistant Enterobacterales have been spreading worldwide (European Centre for Disease Prevention and Control, 2017; Van Duin and Doi, 2017).

Regarding CRKp in our study, the 57.5% NDM carbapenemase rate among the 40 CRKp isolates was higher than the 7%–30% rates reported from the Arabian Gulf region (Al-Zahrani and Alasiri, 2018; Abid et al., 2021; Alraddadi et al., 2022; Alshahrani et al., 2022; Balkhair et al., 2023; Alharbi et al., 2026), 6% – 20% reported from Iran (Solgi et al., 2018; Pourgholi et al., 2022; Soltani et al., 2022), 4%–46% reported from North Africa (Loqman et al., 2021; Ahmed et al., 2023; Taha et al., 2023) and the 3%–24% reported from the European countries (European Centre for Disease Prevention and Control, 2017; Van Duin and Doi, 2017). However, in Gaza and Tunis, the reported rates were higher (86% and 89%, respectively) (Zouaoui et al., 2023; Laham et al., 2025).

On the other hand, the 22.5% OXA carbapenemase rate among the CRKp in our study was lower than the 33%–82% rates reported from almost all the above-mentioned countries except for the lower reported rates from Jerusalem (9%) (Abu Hadba et al., 2025), Greece (3%) (Tsolakidou et al., 2025), and Italy (4.5%) (Bianco et al., 2022), and other European countries (European Centre for Disease Prevention and Control, 2017; Van Duin and Doi, 2017). However, an almost similar OXA rate to ours was reported from Romania (21%) (Lazar et al., 2024).

Fluctuating rates among CRKp carbapenemases were reported by Hussein et al. (2022) at the Rambam Health Care Campus in Haifa between the years 2014 and 2020, indicating a decrease in KPC from 100% to 56%, while showing an increase of OXA from 4% to 29% and NDM from 2% to 12.7%.

The dual co-expression/production rate of different carbapenemases in a single isolate of CRE producers has been reported in several publications from different countries, as shown below. In our study the dual co-expression rate of carbapenemases among the CRKp was 12.5%, exclusively as a combination of NDM + OXA. This pattern was also reported with different frequencies from Saudi Arabia (4-20%) (Al-Zahrani and Alasiri, 2018; Alraddadi et al., 2022; Alshahrani et al., 2022; Alharbi et al., 2026), Qatar (8%) (Abid et al., 2021), Oman (17%) (Balkhair et al., 2023), Egypt (26%) (Ahmed et al., 2023), Turkey (26%) (Genç et al., 2022), Greece (11%) (Tsolakidou et al., 2025), Iran (36%) (Solgi et al., 2018), Morocco (34%) (Loqman et al., 2021), and Romania (49%) (Lazar et al., 2024), but not detected in Italy (Bianco et al., 2022).

The absence of carbapenemases detection was reported in fluctuating rates among CRKp isolates from different countries. In our study, it was 7.5% which differs from those reported from Qatar (15%) (Abid et al., 2021), Oman (1%) (Balkhair et al., 2023), Iran (9% –11%) (Pourgholi et al., 2022; Soltani et al., 2022), Morocco (10%) (Loqman et al., 2021) and Gaza (5%) (Laham et al., 2025).

Regarding the 100 characterized CREc isolates in this study, only two carbapenemase types were detected, predominated by NDM (64%), followed by OXA (29%). Co-carbapenemase production was detected only in one isolate (NDM+OXA), and no carbapenemases were detected in 6% of these CREc.

The findings of CREc, in our study, were compared with other studies from Lebanon, regional, and other countries. In Lebanon, our data differ from the North Lebanon study involving only 33 CREc isolates recovered from 3 different hospitals during 2019 and 2020, which reported lower prevalence of NDM (52%) and higher prevalence for OXA (48%) (Rima et al., 2022).

In comparison to regional studies, the carbapenemases among our isolates (64% NDM and 29% OXA) showed higher rates in NDM and lower rates in OXA, respectively, compared to those isolates reported from Qatar (35% and 55%) (Abid et al., 2021), North Lebanon (54% and 46%) (Rima et al., 2022), Spain (4% and 73%) (Cañada-García et al., 2022), and Italy (18% and 43%) (Bianco et al., 2022).

The 1% co-expression of NDM+OXA carbapenemases detected in our study was lower than those reported from Qatar (3%) (Abid et al., 2021) and Morocco (23%) (Loqman et al., 2021), but almost similar to those reported from Italy (2%) (Bianco et al., 2022).

The absence of carbapenemase detection among CREc in our study was 6%, being higher than the 3% reported from Qatar (Abid et al., 2021) but lower than the 15% reported from Morocco (Loqman et al., 2021). Several possible factors can be involved in explaining such a finding including the limitation of the used kit in detecting variants, other enzymes (e.g., GES, IMI, SME), or having different mechanisms of resistance such as ESBL plus porin loss, AmpC hyperproduction, efflux pump upregulation, or a combination of these mechanisms (Cerqueira et al., 2017).

Historically at our medical center, the first *bla*OXA was detected in CRKp recovered from an 8- year-old pediatric case with urinary tract infection in 2008 and started to spread thereafter (Matar et al., 2008; Matar et al., 2010). In 2012, *bla*NDMs were detected in CREc and CRKp coming from Iraqi patients seeking medical treatment at our center (El Herte et al., 2012a). The spread of these pathogens alerted the medical community and prompted conducting several studies to understand their phenotypic and genotypic characteristics, antimicrobial profiles, and mechanisms of resistance (El Herte et al., 2012b; Tokajian et al., 2016; Araj et al., 2018; Bitar et al., 2018; Sobh et al., 2024).

The exact reason for the variable rates and divergent patterns of carbapenemases is not exactly delineated. However, many factors can be assumed, including differences in antimicrobial usage and selection pressure, infection control capacity and practices, access to advanced or proper diagnostics, and country origin of the patients. For example, residents or patients originating from the Indian subcontinent show a high prevalence of NDM (Boyd et al., 2020). Travel and medical tourism have frequently been implicated in the international importation of multidrug-resistant pathogens (Van Duin and Doi, 2017).

The effect of carbapenemase types regarding response to antimicrobial agents among CRKp and CREc has been addressed for better understanding in patients’ care. In general, it was revealed that plasmids carrying the *bla*NDM can cause multiple drug resistance by harboring other carbapenemases and ESBL genes; thus, MBL-producing isolates cause higher resistance than OXA- and KPC- producing strains (Nordmann et al., 2011).

In our study, similar findings were observed, whereby isolates with positive NDM carbapenemases showed generally higher resistance, reflected in the high MIC values against the three tested carbapenems compared to isolates with OXA carbapenemases among both CRKp and CREc isolates (**Table 3**). Moreover, the CREc with positive OXA carbapenemases showed generally higher susceptibility to the tested antimicrobial agents compared to CRKp isolates with positive OXA carbapenemases (**Table 3**). Such findings were also reported in the literature (Poirel et al., 2012).

Regarding the activity of routinely tested antimicrobial agents against the CRKp and CREc isolates, in our study, poor susceptibility results were revealed (**Table 2**). As shown, the activities of, piperacillin/tazobactam, ciprofloxacin, and trimethoprim-sulfamethoxazole were generally very low to negligible (0%-44%) especially against NDM carbapenemases. However, tigecycline, fosfomycin, and amikacin retained good activity against NDM-and OXA-producing CREc, with variable activity against CRKp. Similar findings, with some variation, were reported from Egypt and Jerusalem (Abu Hadba et al., 2025).

Because of the weak activity of the regular antibiotics, novel antimicrobial agents were introduced to aid in the treatment of the critical CR strains of Enterobacterales and others (Doi, 2019). At our medical center, CZA has been introduced since August 2023. In the present study, CZA activity was nil against NDM carbapenemases in both species but showed acceptable activity against OXA carbapenemases in CREc (86%) and CRKp (78%), aligning with what was reported in the literature (Nordmann et al., 2023). However, our data was lower than the 93% for CREc and 98.7% for CRKp reported earlier by the ATLAS 2018–2021 program, and from the 95.2% susceptibility rate of the CRKp with OXA carbapenemase producer reported from Turkey (Ozger et al., 2021; Lee et al., 2024). It is worth noting that the long use of CZA led toa change in the pattern of carbapenemases in CRE from *bla*KPC to *bla*VIM predominance as was reported from Greece. Thus, one should be cautious and judicious in the use of CZA to avoid or minimize the risk of selection of MBL producers, especially in places where MBLs are predominant (Papadimitriou-Olivgeris et al., 2019).

Since the most common carbapenemases detected in the current study were NDM and OXA, effective antimicrobial therapy options are very limited, and their management remains particularly challenging, especially in the absence of available novel antimicrobial agents in the country (Boyd et al., 2020). In Lebanon, β-lactam/β-lactamase inhibitor combination drugs other than CZA, such as Imp-R and Mer-V, in addition to CFD, have not been introduced so far. Thus, our study documented the effectiveness of these novel antimicrobial agents against the types of carbapenemases produced by CRKp and CREc (**Table 3**), setting a baseline for future comparison.

In the present study, Imp-R susceptibility among CREc was 43% against OXA carbapenemases and 0% against NDM, whereas among CRKp, it was 6% against OXA and 0% against NDM. On the other hand, variable intermediate susceptibility values were noted (34%–49%) for Imp-R against OXA in both species (**Table 3**). These percentages are lower than those reported from the North Lebanon study and other countries (O'Donnell and Lodise, 2022; Nordmann et al., 2023; Santerre Henriksen et al., 2024).

Mer-V does not inhibit MBLs and has a fluctuating effect against isolates with OXA enzymes, as shown in our study and those from France and Taiwan (Lomovskaya et al., 2017; Kuo et al., 2021; Petillon et al., 2025). In the current study, Mer-V activity against OXA was higher vs NDM among isolates of both CREc (96% vs 10%) and CRKp (50% vs 6%). These higher susceptibility findings of Mer-V for OXA were similar to what was reported in the ATLAS 2018–2021 study, which showed that the Mer-V susceptibility was 59.7% and 17.1% for OXA-producing CREc and CRKp. The low CRKp susceptibility rate to Mer-V could be related to co-carriage of ESBL genes. Other studies from Europe showed similar findings (Lee et al., 2024). These data indicated that the higher CREc OXA susceptibility can reflect the weaker carbapenemase activity of OXA in *E*. *coli*, which may still permit meropenem’s intrinsic activity even when vaborbactam cannot inhibit OXA directly. The very low susceptibility against NDM in both species is entirely consistent with the isolate mechanisms and international literature. CFD is one of the few approved agents with inherent activity against NDM-producing Enterobacterales (Zhanel et al., 2019; Viale et al., 2023; Santerre Henriksen et al., 2024). In our study, CFD showed high activity against CREc being 93% against OXA-producing and 72% against NDM-producing isolates, while among the CRKp, it was 100% against OXA-producing isolates and 60% against NDM-producing isolates. Furthermore, intermediate susceptibility values of CFD against NDM carbapenemases ranged from 18%–21% in both species. In line with our findings, the North Lebanon study reported a cefiderocol susceptibility of 85% across combined CRKp and CREc isolates (Rima et al., 2022). Additionally, a report from Gaza on pediatric CRKp indicated CFD was the only agent achieving susceptibility (55%) that could be considered for empirical treatment (Laham et al., 2025). Such findings are in line with the study of O’Donnell and Lodise, which reported that only CFD but no other tested β-lactam/β-lactamase inhibitor was active against NDM enzymes (O'Donnell and Lodise, 2022). Resistance to CFD is associated with a combination of mechanisms, including ESBL/AmpC combinations; mutations affecting function and expression of siderophore receptors, porins, and/or efflux pumps; penicillin-binding protein 3; hetero-or cross-resistance; and co-selection (Karakonstantis et al., 2022). Transmission prevention of carbapenemase-producing Enterobacterales among patients is important and requires coordinated efforts and approaches, including rapid identification and characterization of these resistant pathogens; antimicrobial stewardship programs to reduce the overuse and misuse of antimicrobial agents, especially avoiding certain antibiotics, such as carbapenems, which are direct drivers of carbapenem resistance; cohort isolation of patients infected with these pathogens harboring carbapenemases; implementation of strict infection prevention and control practices and measures; and enforcement of screening of patients upon hospital admission to detect CRE carriage in order to prevent nosocomial transmission. The strength of our study is that for the first time in Lebanon, a comprehensive prospective study was conducted to characterize a substantial number of CREc and CRKp with regard to phenotypes of carbapenemases and their antimicrobial susceptibility against novel and routinely tested antimicrobial agents. AUBMC is considered the largest tertiary care medical center in Lebanon and treats patients, especially complicated cases, from all over the country. Its clinical microbiology laboratory has also been accredited by the College of American Pathologists since 2004.

A limitation of this study was its confinement to *in vitro* testing, as it would have been more clinically informative if patients’ detailed medical histories were also taken into consideration. Though AUBMC is a major tertiary care center in Lebanon, the reported findings can be biased and cannot exactly reflect the status of such pathogens in this country. Further, though the Carba-5 kit used has high sensitivity (99.2%) and specificity (100%), studies with molecular analysis remain the reference method and would have been warranted to help reveal epidemiologic aspects and trace hospital and community transmission. This would hopefully be possible to pursue in the future, as such a study requires funding, which is beyond our capacity, especially during the current devastated economic situation Lebanon is passing through.

## 5 Conclusion

This is the first comprehensive *in vitro* study from Lebanon revealing high resistance of novel antimicrobial agents that have not yet been introduced in the country against the different carbapenemase types encountered among CR Enterobacterales. This highlights the necessity of scrutinizing the proper use of antimicrobial agents and stewardship programs, avoiding the deleterious impact of resistant pathogens. Also, effective nosocomial transmission control of such critically resistant pathogens requires a multidisciplinary approach incorporating rapid detection and characterization of the pathogens, ongoing surveillance, and strengthening and implementation of antimicrobial stewardship and infection control programs to preserve novel therapeutic options for these resistant pathogens.

## Data Availability

The original contributions presented in the study are included in the article/supplementary material. Further inquiries can be directed to the corresponding author.
